# Evaluation of Commercial Disinfectants against *Staphylococcus lentus* and *Micrococcus* spp. of Poultry Origin

**DOI:** 10.1155/2020/8811540

**Published:** 2020-09-25

**Authors:** Otun Saha, Nadira Naznin Rakhi, Arif Istiaq, Israt Islam, Munawar Sultana, M. Anwar Hossain, Md. Mizanur Rahaman

**Affiliations:** ^1^Department of Microbiology, University of Dhaka, Dhaka-1000, Bangladesh; ^2^Department of Biotechnology and Genetic Engineering, Bangabandhu Sheikh Mujibur Rahman Science and Technology University, Gopalganj, Bangladesh; ^3^Department of Developmental Neurobiology, Graduate School of Medical Sciences, Kumamoto University, Kumamoto, Japan; ^4^Jashore Science and Technology University, Jashore, Bangladesh

## Abstract

**Introduction:**

Effective sanitation strategies for poultry farms require an appropriate selection of the disinfectant based on the contaminants present and their sensitivity to the disinfectants.

**Aim:**

The current study investigated the prevalence of streptococci/micrococci in poultry farms of Bangladesh and the efficacy of commercial disinfectants (Savlon, Lysol, Quatovet, Virkon S, and Virocid) along with alcohol against these pathogens to adopt appropriate strategies.

**Materials and Methods:**

Conventional approaches and the 16S rRNA gene sequencing were performed to confirm the isolates at the species level along with microtiter biofilm assay to determine their biofilm-forming ability. Efficacy of the disinfectants was tested against those isolates using agar well diffusion and minimum inhibitory concentration (MIC) test by broth dilution method using different dilutions of the disinfectants.

**Results:**

*Staphylococcus lentus* (*n* = 32), *Micrococcus luteus* (*n* = 7), and *Micrococcus aloeverae* (*n* = 4) were confirmed among 102 presumptively screened streptococci/micrococci isolates from 43 samples. No single disinfectant showed equally high efficacy against all three bacterial species in agar well diffusion test, although Virocid showed the lowest MIC against all three of them. Lysol was least effective among the commercial disinfectants by both MIC and diffusion method, although each commercial disinfectant was more effective than alcohol. Considering both the average diameter of the inhibition zones and the MIC values, efficacy can be interpreted as Virocid > Quatovet > Savlon > Virkon S > Lysol. Although the efficacy decreased with decreasing concentration, the disinfectants retained a satisfactory level of efficacy at 50% concentration. Among test pathogens, *M. aloeverae* was the most sensitive to the disinfectants and the weakest biofilm producers, whereas 4/14 *S. lentus* and 1/5 *M. luteus* were strong biofilm producers, which may cause more reduction in the efficacy in environmental conditions.

**Conclusion:**

As no ideal disinfectant was found in the study, the efficacy of the disinfectants should be routinely evaluated and validated to ensure the sanitation standards in the poultry sector.

## 1. Introduction

Poultry farming is one of the most promising and fastest growing agrobased enterprises in Bangladesh contributing about one-third of total GDP (18.6%) from the agricultural sector of the country [1, 2]. Currently, it is even growing at a rate of 20% per annum [2]. Besides, poultry is one of the largest domestic animal stocks globally including Bangladesh [2, 3]. So, ensuring biosecurity for poultry is mandatory for economic prosperity and public health. But unfortunately, the manpower comprised of more than 3 million people associated directly with this sector [4] is mostly unskilled [5] and lacking proper knowledge of personal hygiene and sanitation strategies, thus risking this promising sector. Moreover, the lack of sufficient knowledge and training on the sanitation program is making the zoonotic disease transmission difficult to control/prevent leading to high health risks for both animals and farmers [6].

The recent reports showed that most of the poultry farms do not practice the benchmark guidelines of biosecurity [7]. Spraying disinfectants in sheds and removing feces were the only sanitation schemes adopted in the farms [8, 9]. Even those disinfectants are used without regular validation and evaluation of efficacy, while the efficacy of the disinfectants is influenced by formulation, level of organic load, humidity, temperature, dilution rate, pH and hardness of water, and other factors [9, 10]. Also, using disinfectants without validation and evaluation may cause the high selective pressure leading to the gradual decrease in sensitivity of the organisms to the disinfectants used and even cross-resistance to antibiotics of public health concerns [11]. Thus improper sanitation procedures might be ineffective in disease control lowering bird performance [11]. So, the evaluation of the disinfectants' efficacy should be in priority to select the suitable disinfectant by minimizing the microbial load before slaughtering and processing of the carcasses.

Gram-positive organisms including *Micrococcus* spp., *Corynebacterium* spp., and *Staphylococcus* spp. are predominant in poultry microflora [12] and prevalently found in barns [13], in entry area [14], and even in bioaerosol derived from animal dander and feces of the poultry houses [15]. *Micrococcus* spp., *Enterococcus* spp., and *Staphylococcus* spp. are also found predominating on eggshells [15, 16], while eggshells are used in the poultry feeds as the source of calcium [17]. So, this practice along with lack of personal hygiene practices among the farm workers observed in the previous studies [8], which included no practice of disinfecting hands and feet before entering and exiting and even eating and touching eyes, faces, bodies, and clothing without washing hands, etc., denotes the potential of health hazards in both poultry and poultry farm workers. Besides, *Staphylococcus aureus, S. xylosus, S. lentus, S. auricularis, S. hominis, Bacillus cereus,* and *Micrococcus* spp. [15] are considered as the main issues of meat safety due to faulty implementation of Hazard Analysis Critical Control point (HACCP) rather than the poultry pathogens [18]. However, although *Staphylococcus* spp. are not considered as one of the prime poultry pathogens, they are associated with different infections in birds, especially the coagulase positive *Staphylococcus* spp. and *S. aureus* as well as coagulase-negative staphylococci [19]. Among coagulase-negative staphylococci, *S. xylosus, S. lentus*, and *S. cohnii* are predominating and associated with central nervous system of poultry [20]. On the other hand, *Micrococcus* spp. are generally considered as nonpathogen, and *Micrococcus pyogenes* var. *albus* and *M. candidus* were isolated from birds with aerosaccitis disease [21] and also reported to cause secondary infections [15, 21]. Moreover, *Micrococcus* is one of the dominant species found in the particulate matters of poultry houses that can cause different diseases in humans and animals [15].

However, these reports are from the poultry farms worldwide, while the prevalence of micrococci/staphylococci is not well reported from Bangladesh. Besides, although few studies evaluated efficacy of the disinfectants against poultry pathogens, unfortunately, the efficiency of any disinfectant has never been tested against these group of organisms. So, the current study targets the prevalence of these organisms and the in vitro efficacy of commercial disinfectants against these organisms to devise a sanitation program appropriate for the poultry farms of the country.

## 2. Materials and Methods

### 2.1. Ethics Approval

Consents were obtained from the owners of the farms before the samples were collected for the study. Besides, the Ethics Committee of the Faculty of Biological Sciences, University of Dhaka, Bangladesh, approved the procedure, Reference 71/Biol.Scs./2018–2019.

### 2.2. Sample Collection

A total of 43 poultry samples (Cloacal swab, *n* = 19; Liver, *n* = 24) were collected from 3 different poultry farms, with or without disease signs from Manikganj and Narsingdi districts under Dhaka Division, Bangladesh, during the period between 2017 and 2018. After sampling, samples were transferred to the Department of Microbiology, University of Dhaka, within the shortest possible time.

### 2.3. Screening of Micrococcus and Staphylococcus

Samples were inoculated into nutrient broth (Oxoid, USA) followed by pre-enrichment in brain heart infusion (BHI) broth (Oxoid, USA) according to Food and Drug Administration Bacteriological Analytical Manual (FDA-BAM) [22]. Samples were then directly inoculated onto blood agar (Oxoid, USA) with 5% sheep blood and mannitol salt agar (MSA) (Oxoid, USA) for screening of *Micrococcus* spp. and *Staphylococcus* spp., respectively, along with the biochemical tests (triple sugar iron, indole, methyl-red, Voges–Proskauer, citrate, catalase, oxidase, and sugar (glucose) fermentation test) following Bergey's manual of determinative bacteriology [23].

### 2.4. Molecular Identification of the Isolates and Phylogenetic Analyses

Presumptive *Micrococcus* and *Staphylococcus* isolates were genotyped by Random Amplified Polymorphic DNA (RAPD) method using arbitrary primer 1283 (5′- GCGATCCCCA-3′) [24]. 16S rRNA genes of the representative isolates from each RAPD group were amplified by PCR using universal primers 27F (5′-AGAGTTTGATCCTGGCTCAG-3′) and 1492R (5′-CTACGGCTACCTTGTTACGA-3′) and subjected to sequencing [4]. DNA sequencing was carried out using Applied Biosystems highest capacity-based genetic analyzer (ABI PRISM® 377 DNA Sequencer) platforms with the BigDye® Terminator v3.1 cycle sequencing kit chemistry. Raw data were analyzed and assembled followed by submission to NCBI GenBank (MN701067, MN701075, and MN701081). For inferring the evolutionary history of the 16S rRNA gene of the selected isolates, neighbor-joining method [24] was used using MEGA7 [25] based on Kimura-2-parameter model.

### 2.5. Biofilm Assay of Representative Micrococcus and Staphylococcus Isolates

Randomly selected isolates of both *Micrococcus* and *Staphylococcus* were subjected to microtiter biofilm assay [26]. The assay was performed in duplicate in the 96-well tissue culture plates. The observed optical density (OD) was evaluated to determine the biofilm-forming ability of the isolates on a 4-grade scale (nonadherent, weakly adherent, moderately adherent, and strongly adherent) [26, 27]. This 4-grade was determined by comparing OD with ODc (three standard deviation values above the mean OD of the negative control).

### 2.6. Selection of Disinfectants

The commercial disinfectants selected for this study were Lysol (active ingredients: benzalkonium chloride, manufactured by Reckitt Benckiser, Bangladesh), Savlon (active ingredients: cetrimide and chlorhexidine gluconate combination, manufactured by ACI, Bangladesh), Virocid® (active ingredients: combination of quaternary ammonium compounds, glutaraldehyde, and alcohol: isopropanol, pineoil; manufactured by CID LINES, Belgium), Quatovet (quaternary ammonium compound, manufactured by THESEO Deutschland GmbH, Germany), and Virkon S (potassium peroxymonosulfate and sodium chloride) along with locally available alcohol (70% v/v) for laboratory use.

### 2.7. Efficacy Testing of Disinfectant and Correlation of Efficacy with Disinfectant Concentration

The in vitro efficacy testing of the disinfectants against *Micrococcus* and *Staphylococcus* was determined by agar well diffusion [28]. Mueller–Hinton agar (Oxoid™, UK) plates were inoculated with the pure cultures of the selected isolates using sterile cotton swab and five 7 mm wells were made, one in the center and the other four about 20 mm away from the center. 50 *μ*L of each disinfectant was added to the wells followed by overnight incubation to determine the zone of inhibition. To investigate the correlation between the disinfectant efficacy and the concentration, varying concentrations (30%, 40%, 50%, 60%, 70%, 80%, 90%, and 100%) of the disinfectant solutions were prepared and tested for efficacy accordingly. The assay with each concentration was performed in triplicate to determine the selected MIC values of the isolates [28]. MICs of disinfectants in these isolates were determined using broth microdilution as described by Olasehinde et al., 2015 [29].

### 2.8. Statistical Analysis

Descriptive statistics including mean, standard deviation (SD), and the range were used to summarize the antimicrobial properties of all disinfectants using Social Sciences (SPSS) version 21.0 software (IBM, Armonk, NY, USA) [30] and Microsoft Excel.

## 3. Results

### 3.1. Isolation of Staphylococcus and Micrococcus Isolates

**Figure 1:**
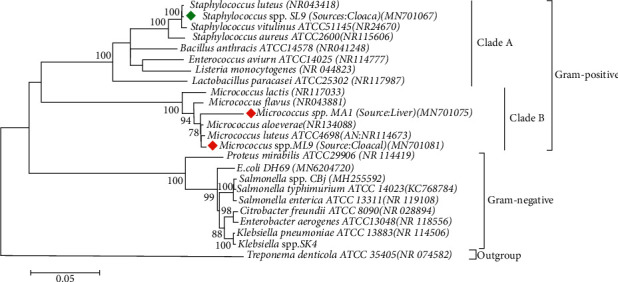
Phylogenetic tree predicted by the neighbor-joining method using 16S rRNA gene sequences. The evolutionary distances were computed using the Kimura 2-parameter model method and are in the units of the number of base substitutions per site. The bootstrap considered 1000 replicates. The scale bar represents the expected number of substitutions averaged over all the analyzed sites. The optimal tree with the sum of branch length = 0.98517644 is shown here. The results obtained with sequencing exhibited two different clusters, and we referred to them as clusters A and B. However, due to the high-sequence similarity, the nodes are not very stable and sequencing errors in some of the older sequences may well affect the topology of the tree. Cluster A contains *Staphylococcus lentus*. Cluster B contains both *Micrococcus luteus* and *Micrococcus aloeverae* strains. *Treponema denticola* was used as outgroup.

Following the inoculation of 43 poultry samples onto mannitol salt agar and blood agar, a total of 80 presumptive *Staphylococcus* isolates and 22 presumptive *Micrococcus* isolates were retrieved, respectively. So, a total of 102 isolates were investigated phenotypically and genotypically to isolate *Staphylococcus* and *Micrococcus.* According to the biochemical test results, 11 out of 22 retrieved isolates from blood agar and 32 out of 80 isolates from mannitol salt agar were presumptively *Micrococcus* and *Staphylococcus*, respectively. Later, RAPD analysis of the isolates differentiated these 43 isolates into 3 different groups (Figure S1). Sequencing of 16S rRNA gene of the representative isolates of each group identified RAPD groups 1, 2, and 3 as *S. lentus* (*n* = 32), *M. luteus* (*n* = 7), and *M. aloeverae* (*n* = 4), respectively (Figure 1). So, out of a total of 102 isolates, *S*. *lentus* was more prevalent (31.37%, 32/102) than *Micrococcus* spp. (10.78%, 11/102). The prevalence of both *Micrococcus* spp. and *Staphylococcus* spp. was higher in cloacal swab samples (72.72%, 8/11 and 59.38%, 19/32, respectively) than in liver samples (27.27%, 3/11 and 40.63%, 13/32, respectively).

### 3.2. Biofilm Assay of the Representative Isolates

**Figure 2:**
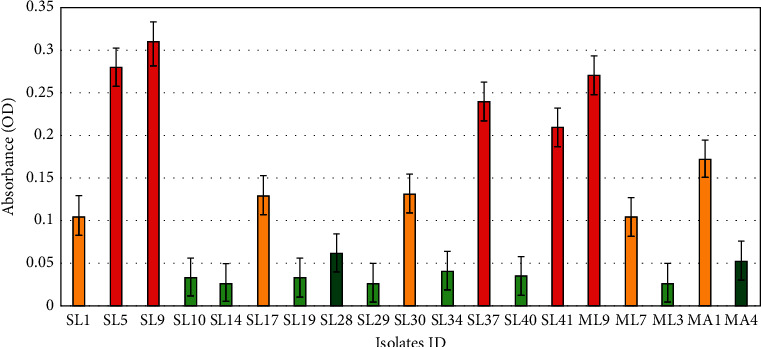
Results of biofilm formation in the microtiter plate assay by individual isolates. Red, yellow, light green, and bottle green indicated the strong, the moderate, the weak, and the lack of biofilm formation ability, respectively. Y-axis represents the numerical values of the absorbance of each isolate, and X-axis represents the isolates. Here, the absorbance values represent the average OD of each isolate. The *S. lentus* strains: SL1, SL5, SL9, SL10, SL14, SL17, SL19, SL28, SL29, SL30, SL34, SL37, SL40, and SL41; the *M. luteus* strains: ML9, ML7, and ML3; and *M. aloeverae* strains: MA1,and MA4 showed OD of 0.106, 0.28, 0.31, 0.034, 0.0273, 0.13, 0.033, 0.062, 0.027, 0.132, 0.041, 0.24, 0.035, 0.21, 0.273, 0.104, 0.028, 0.173, and 0.053, respectively.

Twenty-two isolates (14 *S. lentus*, 5 *M. luteus*, and 3 *M. aloeverae)* were randomly selected and subjected to biofilm assay. Thirteen out of those 22 isolates (59.09%) were found to be biofilm producers. Among the isolates, 28.57% (4/14) of *S. lentus* and 20% (1/5) of *M. luteus* possessed strong biofilm-forming ability, while 21.43% (3/14) of *S. lentus* were found to be moderate biofilm producers (Figure 2 and Table S1). In this study, *M. aloeverae* showed relatively weak biofilm-forming ability compared to others.

### 3.3. In Vitro Efficacy Testing of the Disinfectants

**Figure 3:**
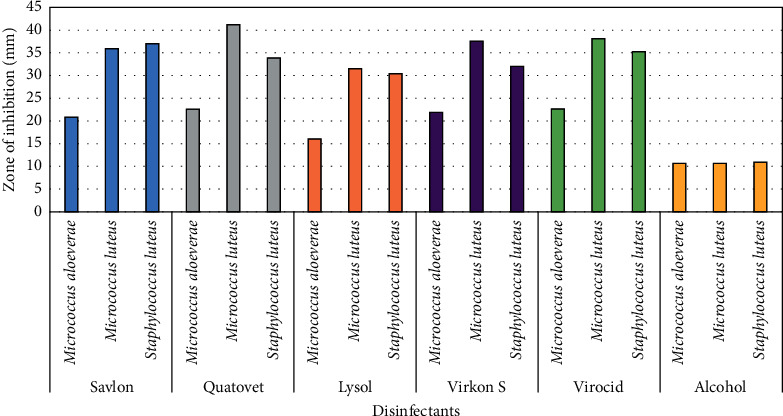
Diagrammatic representation of in vitro efficacy of disinfectants (100% conc.) against Gram-positive bacteria. *Y* axis represents the diameter of inhibition zone (mm). Here, blue, gray, pink, violet, green, and yellow indicate Savlon, Quatovet, Lysol, Virkon, S. Virocid, and alcohol, respectively.

**Figure 4:**
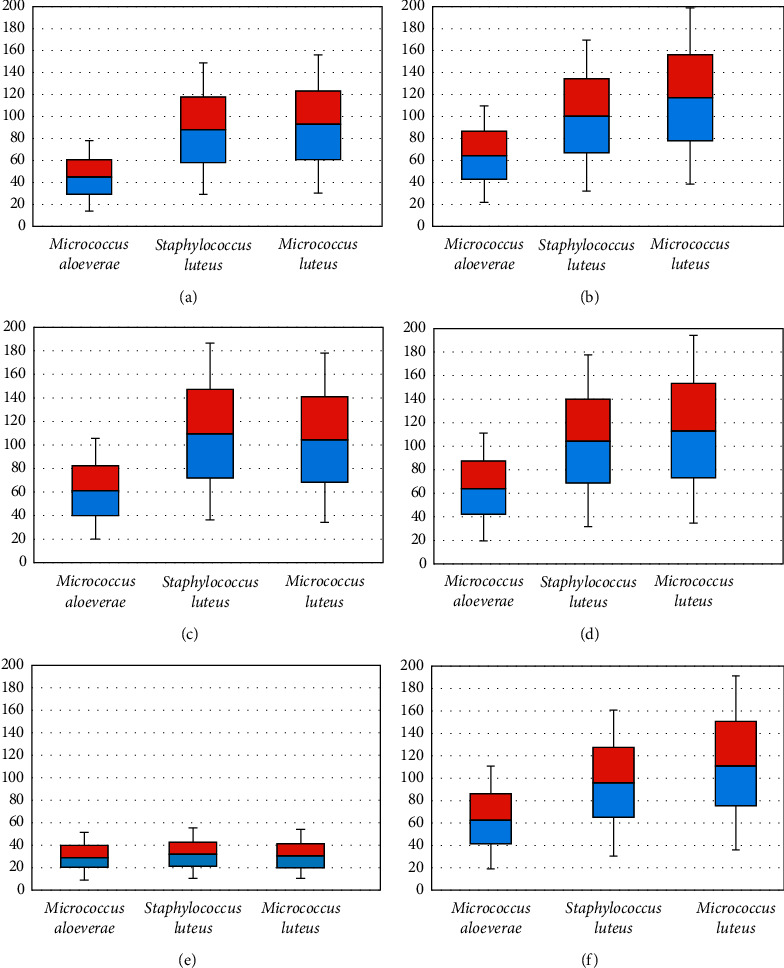
Box and Whisker plot (min/max, lower/upper quartiles and median) showing overall disinfection efficacy of various disinfectants against streptococci/micrococci groups of bacteria. *Y* axis shows the average diameter of inhibition zones. Here, (a) Lysol; (b) Quatovet; (c) Savlon; (d) Virocid; (e) Alcohol; (f) Virkon S.

Agar well diffusion test of 22 selected isolates (*Micrococcus* spp., *n* = 8 and *S. lentus*, *n* = 14) against the disinfectants showed that, except for Virkon S and Savlon, *M. aloeverae* was most sensitive to disinfectants, while *M. luteus* was the least sensitive to all but Virkon S among the three species tested. Besides, Lysol gave the lowest zones of inhibition against all the isolates except *M. aloeverae* (Table 1)). However, all of the commercially available disinfectants were more effective than alcohol (70% v/v) (Figure 3). Savlon showed the highest zone of inhibition (D) (37.13 ± 0.7 mm) for *S. lentus* followed by Virocid (35.25 ± 0.42 mm), Quatovet (33.9 ± 0.67 mm), and Virkon S (32.42 ± 0.4 mm), respectively. In case of *M. luteus*, Virkon S was most effective (*D* = 37.32 ± 0.5 mm) followed by Virocid (*D* = 22.5 ± 0.56 mm), Quatovet (*D* = 21.88 ± 0.51 mm), and Savlon (*D* = 21.13 ± 0.76 mm), while Quatovet (*D* = 40.13 ± 1.18 mm) was found the most effective for *M. aloeverae* followed by Virocid (*D* = 38.63 ± 74 mm), Savlon (*D* = 35.5 ± 0.69 mm), and Virkon S (*D* = 22.1 ± 0.3 mm). So, considering the average of the inhibition zones against both *S. lentus* and *Micrococcus* spp., the efficacy of the commercial disinfectants can be concluded as Virocid > Quatovet > Savlon > Virkon S > Lysol (Table 1). Individually, for *S. lentus*, the efficacy of the disinfectants was observed in descending order as Savlon > Virocid > Quatovet > Virkon S > Lysol, for *M. lentus*, Virkon S > Virocid > Quatovet > Savlon > Lysol, and for *M. aloeverae*, Quatovet > Virocid > Savlon > Lysol > Virkon S (Figure 4). Moreover, Virocid showed the lowest MIC value, 10% (v/v) for each of the bacterial species tested (Table 2). Along with Virocid, Quatovet showed MIC value at 10% in case of both *S. lentus* and *M. luteus.*

### 3.4. Correlation between the Efficacy and the Concentration of Disinfectants

**Figure 5:**
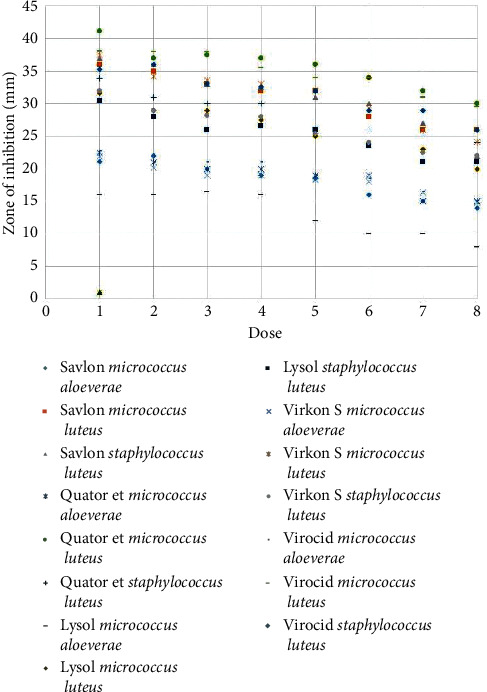
Inhibition zone diameters of selected isolates of micrococci/staphylococci organisms against the disinfectants tested at different concentrations varying from 100% to 30% dose concentrations. *X* axis represents the concentrations and *Y* axis represents the diameters of inhibition zones in millimeter (mm).

Agar well diffusion assay using the varying concentrations of each disinfectant showed that the efficacy of each disinfectant decreased with decreasing concentration (Table 3). But the inhibition zone diameter was of ≥62.5% of the inhibition zone diameter observed at absolute concentration at ≥50% concentration of each commercial disinfectant. However, alcohol failed to produce any inhibition zone at 60% concentration in case of *M. aloeverae,* at 50% concentration in case of *S. lentus*, and at 40% concentration in case of *M. luteus*, while none of the tested isolates showed resistance to any commercial disinfectant up to 30% concentration of the disinfectants (Figure 5).

## 4. Discussion

An effective sanitation plan for biosecurity of the poultry is based on the appropriate selection of disinfectants, which should consider the contaminants present and their sensitivity to the available disinfectants [31]. While among diverse microorganisms of poultry origin, the streptococci-micrococci group of bacteria is one of the predominant contaminants of poultry representing about 35% to 92% of the total poultry contaminants [8, 15, 31], no data for these organisms have been reported from the poultry sector of Bangladesh, let alone the efficacy of the commonly used disinfectants against the organisms. Therefore, the current study targeted this group of poultry microbes and found that *S. lentus* was more prevalent (32/102, 31.37%) than micrococci including *M. luteus* (7/102, 6.86%) and *M. aloeverae* (4/102, 3.9%). Such a difference in the prevalence of staphylococci and micrococci was previously reported in another study [32]. Moreover, *S. lentus* is frequently reported to be isolated from poultry sources along with other coagulase-negative staphylococci [20, 33, 34]. However, they are not considered prominent poultry pathogens, although they are associated with various lesions in commercial poultry flocks [35]. But the most threatening concern of staphylococci from animal sources is to transfer antibiotic resistance genes to human pathogens [20] as well as dissemination of the disinfectant resistance genes [11]. The antibiogram results of *S. lentus* isolates showed that the highest resistance was observed against chloramphenicol followed by erythromycin and doxycycline, respectively (Supplementary Table 1). Although the use of erythromycin in poultry feeds was banned in the past due to the reports of significantly higher resistance among staphylococci of animal origin compared to those of human origin [34, 36], the antibiogram result indicated the possible breach of that regulation by relevant personnel. On the contrary, 100% sensitivity to imipenem, cefepime, and gentamycin was observed (Table S1). However, *S. lentus* of animal origin may also pose the risk of zoonosis considering the report of peritonitis in a peritoneal dialysis patient caused by the organism of animal origin [33].

On the other hand, *M. luteus* and *M. aloeverae* which were found in the study were initially thought of as nonpathogens, but they are now regarded as conditional pathogens [37]. While *M. aloeverae* is mostly nonpathogenic with rare reports of virulence [38] and usually not reported from poultry samples, *M. luteus* was found to be associated with pneumonia [38], endocarditis [37], septic arthritis [38], etc. in humans. Notably, opportunistic *M. luteus* was responsible for causing morbid diseases in fish [39]. So, the common practice of using poultry feces as fish feeds/fertilizer without any prior treatment may also contribute to the dissemination of diseases in fish [8]. Besides, this practice along with using eggshells as the calcium supplement in poultry diet [17] may be partially responsible for the high prevalence of antibiotic resistance in human/animal and environmental isolates. However, all micrococci isolates in this study were sensitive to azithromycin and trimethoprim in addition to imipenem, cefepime, and gentamycin like staphylococci isolates, although resistance to doxycycline was observed (Figure S2 and Table S1). Apart from antibiotics, all of the streptococci and micrococci isolates were challenged against frequently used commercial disinfectants (Savlon, Virocid, Lysol, Quatovet, and Virkon S) along with alcohol, as different commercially available disinfectants are alcohol based. Each of these disinfectants was of different active compounds, although Virocid (5th generation) and Quatovet (3rd generation) have quaternary ammonium compound (QAC) in common with the rest of the components differing from each other. Efficacy tests revealed that no single disinfectant was equally effective against all three species tested in both broth dilution and agar well diffusion tests, although commercial ones were more effective than alcohol. While alcohol gave the smallest zones of inhibition against the test isolates, the total absence of inhibition zone in presence of alcohol was previously reported [40]. Besides, similar to our findings, the efficacy of alcohol based disinfectants tested by the same method was also reported below the satisfaction level previously [31]. Hence, the reason could probably be a limitation of the testing method itself, as alcohol, being volatile, might have evaporated before it got into contact with the test organisms. However, followed by alcohol, benzalkonium chloride based Lysol was found least effective, although this class of disinfectant has been reported to be effective previously [40]. Even Lysol was reported to be more effective than Savlon against micrococci [40], which was quite opposite to our findings according to both methods applied.

On the other hand, comparing the size of inhibition zones against three species tested, QAC based Quatovet was found to be one of the best choices for disinfectants in Bangladesh, while resistance to Quatovet was observed in Egypt due to its prolonged use in poultry [41]. The efficacy of the other QACs containing disinfectant, Virocid, slightly differed from Quatovet in both methods. That was probably due to the differences in synergy of QACs with other constituents of those disinfectants. Virkon S was found quite effective especially in the case of *S. lentus* and *M. luteus*. However, despite the limitations of agar well diffusion method including dependence of test efficiency on characteristics, properties, and composition of agar media, solubility of disinfectants and diffusion ability of disinfectant solutions on agar media, lacking the ability to correlate disinfectants' efficacy with exposure time, etc. [31], the results were found in excellent or good agreement with the findings of MIC determined by the broth dilution method. Besides, to validate the findings, the common poultry pathogens such as *Salmonella*, *Klebsiella*, and *E. coli* retrieved from laboratory depository were also challenged against the disinfectants. While Gram-negative organisms tend to be more resistant to disinfectant compared to Gram-positive organisms [40,42], disinfectants showed slightly different efficacy pattern from that obtained with Gram-positive organisms (Figure S3–S5). Quatovet was found most potent against all three Gram-negative isolates followed by Virkon S, Savlon, and Virocid (Table S2). But similar to the efficacy testing with Gram-positive isolates, alcohol followed by Lysol was found least effective.

However, diluting disinfectants with water is a common practice in most of the poultry farms of Bangladesh to make the sanitation programs economically more feasible. But the efficacy of the disinfectants depends on the correct dilution [31]. Efficacy usually decreases with decreasing concentration of disinfectants, although a satisfactory level of efficacy was noticed up to 30% of disinfectant concentration. Besides, at 30% disinfectant concentration, Virocid retained even ≥81% inhibition zone diameters of those obtained at absolute concentration (*x*) indicating the lowest effect of dilution on the efficacy followed by Quatovet (*x* ≥ 78%) and Savlon (*x* ≥ 76%), respectively (Table 3). Lysol showed the highest deviation of efficacy with dilution (*x* ≥ 62.5%). Virkon S, Virocid, and Quatovet showed the least fluctuation in the efficacy with decreasing concentration in case of *M. aloeverae*. But unfortunately, storage before application, exposure to UV, or extreme temperature reduce the efficacy of the diluted disinfectants notably [9], while these field effects were not simulated during the study. So, the same level of efficacy of the diluted disinfectants cannot be claimed at the field level. On the other hand, the gradual decrease in the efficacy of Virkon S with decreasing concentration should be considered carefully, as the higher dose of Virkon S has been reported to interfere with the hatchability and survivability of chicks [42]. Besides, biofilm interferes with the efficacy of the disinfectants making the organisms 1000 times more resistant to disinfectants [9]. On top of that, biofilm-forming property is the major virulence factor and prime cause of multidrug resistance in *S. lentus* [35], while isolates of *S. lentus* in this study included both strong and moderate biofilm producers. On the other hand, only 1 isolate of *M. luteus* was strong biofilm producer and all *M. aloeverae* were weak biofilm producers, although in vitro monoculture of micrococci might show lesser biofilm-forming ability compared to their ability at natural environment because of the association with other species [9]. However, the disinfectants showed that the organism dependent pattern of efficacy. *M. luteus* was least sensitive to all of the disinfectants followed by *S. lentus* and *M. aloeverae*, respectively, although none of the isolates were resistant to any of the disinfectants tested.

However, the study is not denying its limitations, as the in situ performance of the disinfectants might show slight difference from these in vitro findings depending on the environmental and other factors. Nevertheless, each of the test results was validated by ensuring reproducibility.

## 5. Conclusions

Quaternary ammonium compounds based disinfectants, Quatovet, Virocid, Virkon S, and chlorhexidine gluconate-cetrimide based Savlon, were found more potent than benzalkonium chloride based Lysol and Alcohol against staphylococci and micrococci, the predominate contaminants of poultry. Due to frequent fluctuations in the efficacy and emergence of the resistance against disinfectants, sanitation programs should be based on the current evaluation of disinfectants' efficiency against the microflora present in the facilities. So, the poultry industry, being a promising economical sector of Bangladesh, should be taken into consideration for the more vigorous analysis of the microflora and their sensitivity to the disinfectants rendering disinfection program to be more effective and economically feasible.

## Figures and Tables

**Table 1:** Size of the zones of inhibition (mean ± SD) of the organisms produced against each of the disinfectants tested (all data are presented in mm units).

Organisms	Savlon	Virocid	Lysol	Virkon S	Quatovet	Alcohol
RAPD group 1: *Staphylococcus lentus*	37.13 ± 0.7	35.25 ± 0.42	30.13 ± 0.88	32.42 ± 0.4	33.9 ± 0.67	11.13 ± 0.57
RAPD group 2: *Micrococcus luteus*	21.13 ± 0.76	22.5 ± 0.56	15.75 ± 0.74	37.32 ± 0.5	21.88 ± 0.51	10.5 ± 0.69
RAPD group 3: *Micrococcus aloeverae*	35.5 ± 0.69	38.63 ± 0.74	31.38 ± 0.82	22.1 ± 0.3	40.13 ± 1.18	11.00 ± 0.61

**Table 2:** Minimum inhibitory concentration (MIC) values of the disinfectants as determined for *S. lentus* and *Micrococcus* spp. by broth dilution.

Disinfectants	Savlon (%)	Virocid (%)	Lysol (%)	Virkon S (%)	Quatovet (%)
Bacterial group					
*Staphylococcus lentus*	20	10	30	10	10
*Micrococcus luteus*	30	10	40	20	10
*Micrococcus aloeverae*	40	10	50	60	20

**Table 3:** Average diameter of the inhibition zones at different concentrations of the disinfectants tested against *Staphylococcus lentus* and *Micrococcus* spp.

Disinfectants	Organisms	Dose 100%	Dose 90%	Dose 80%	Dose 70%	Dose 60%	Dose 50%	Dose 40%	Dose 30%
Savlon	*Micrococcus aloeverae*	21.1	22	20	19	18.5	16	15	14
	*Micrococcus luteus*	36	35	33	32	32	28	26	26
	*Staphylococcus lentus*	37	36	33	32.5	31	30	27	22

Quatovet	*Micrococcus aloeverae*	22.5	21	20	20	19	19	15	15
	*Micrococcus luteus*	41.1	37	37.5	37	36	34	32	30
	*Staphylococcus lentus*	33.9	31	30	30	26	26	25.5	24

Lysol	*Micrococcus aloeverae*	16	16	16.5	16	12	10	10	8
	*Micrococcus luteus*	31.7	29	29	27.5	25	24	23	20
	*Staphylococcus lentus*	30.5	28	26	26.5	26	23.5	21	21

Virocid	*Micrococcus aloeverae*	22.8	21	21	21	19	18.5	16.5	15
	*Micrococcus luteus*	38.1	38	38	35.5	34	34	31	29.5
	*Staphylococcus lentus*	35.2	36	33	32.5	32	29	29	26

Alcohol	*Micrococcus aloeverae*	10.5	9	9	10	0	0	0	0
	*Micrococcus luteus*	10.8	10.5	10.5	10.5	10	8	0	0
	*Staphylococcus lentus*	11	12	12.5	12	12	0	0	0

All the data are presented as unit, mm.

## Data Availability

All data used to support the findings of this study are included within the article.

## Supplementary Materials

Supplementary Materials Figure S1: gel electrophoretograms image of RAPD typing. Figure S2: diagrammatic representation of antibiotic resistance pattern of the isolates. Figure S3: diagrammatic representation of in vitro efficacy of the disinfectant (100% conc.). Figure S4: Box and Whisker plot (min/max, lower/upper quartiles, and median) showing overall disinfection efficacy of various disinfectants against the Gram-negative bacteria. Figure S5: percentage of the resistance of isolated Gram-negative microorganisms against specific disinfectant at 100% to 30% dose conc. Table S1: antimicrobial susceptibility of the isolates as suggested by CLSI guideline. Table S2: zone of inhibition size (mean ± SD) descriptive statistics of the tested Gram-negative organisms for the disinfectant comparison.
