# Supplementary material for: Evaluation of Commercial Disinfectants against Staphylococcus lentus and Micrococcus spp. of Poultry Origin
Source: Vet Med Int. 2020 Sep 25;2020:8811540. doi: 10.1155/2020/8811540 (PMC7533021; doi:10.1155/2020/8811540)
Supplement: Supplementary Materials — Figure S1: gel electrophoretograms image of RAPD typing. Figure S2: diagrammatic representation of antibiotic resistance pattern of the isolates. Figure S3: diagrammatic representation of in vitro efficacy of the disinfectant (100% conc.). Figure S4: Box and Whisker plot (min/max, lower/upper quartiles, and median) showing overall disinfection efficacy of various disinfectants against the Gram-negative bacteria. Figure S5: percentage of the resistance of isolated Gram-negative microorganisms against specific disinfectant at 100% to 30% dose conc. Table S1: antimicrobial susceptibility of the isolates as suggested by CLSI guideline. Table S2: zone of inhibition size (mean ± SD) descriptive statistics of the tested Gram-negative organisms for the disinfectant comparison. [file 8811540.f1.docx]

Evaluation of commercial disinfectants against *Staphylococcus lentus* and *Micrococcus* spp. of poultry origin

Otun Saha ^1^, Nadira Naznin Rakhi ^2^, Arif Istiaq ^3^, Israt Islam^1^, Munawar Sultana ^1^, M. Anwar Hossain ^1,4,+^ and Md. Mizanur Rahaman ^1,*^

^1^ Department of Microbiology, University of Dhaka, Dhaka 1000, Bangladesh.

^2^ Department of Biotechnology and Genetic Engineering, Bangabandhu Sheikh Mujibur Rahman Science and Technology University, Gopalganj, Bangladesh.

^3^ Department of Developmental Neurobiology, Graduate School of Medical Sciences, Kumamoto University, Kumamoto, Japan.

+Present Position: Vice-Chancellor, Jashore Science and Technology University, Bangladesh.

***** Correspondence: razu002@du.ac.bd; +8801796585290


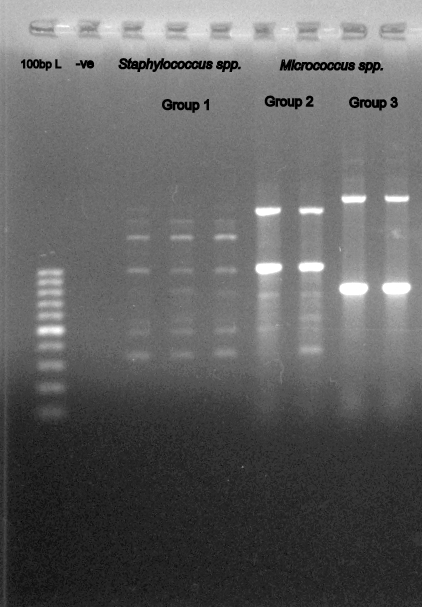


Supplementary Figure 1. Gel electrophoretograms is image of RAPD typing. (Lane 1: 100bp ladder as molecular size DNA marker. Lane 2: -ve control, Lanes 3, Lane 4, Lane 5 represent group 1 (*Staphylococcus lentus*), Lane 6, Lane 7 represent group 2 (*Micrococcus luteus*), Lane 8 and Lane 9 represent group 3 (*Micrococcus aloeverae*).


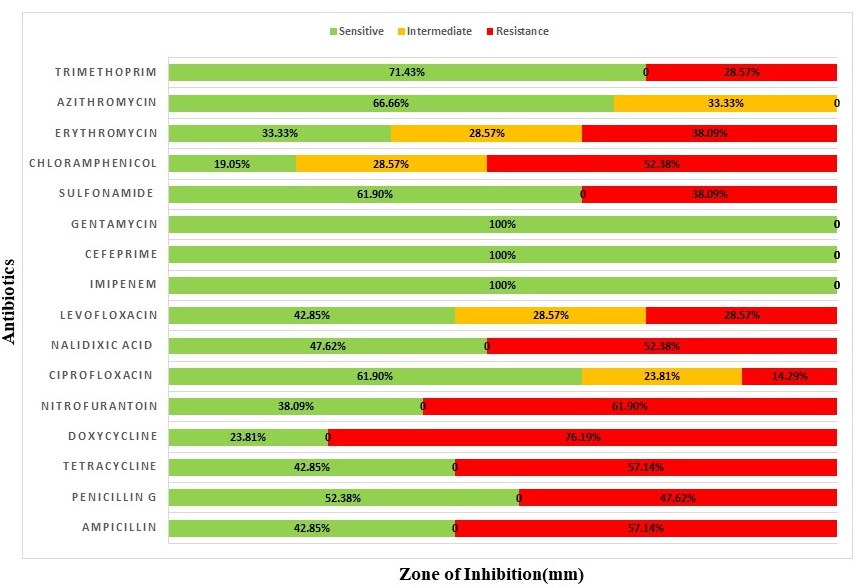


Supplementary Figure 2. Diagrammatic representation of antibiotic resistance pattern of the isolates. AMP= Ampicillin, P= Penicillin G, Te= Tetracycline, DO= Doxycycline, F= Nitrofuran CIP= Ciprofloxacin, Na= Nalidixic acid, Lev= Levofloxacin, IMP= Imipenem, FEP= Cefeprime, CN= Gentamycin, S3= Sulfonamide, C= Chloramphenicol, E= Erythromycin, Atm= Azithromycin, and Tm= Trimethoprim. Green indicated sensitive isolates, Yellow indicated intermediate stage and red shows resistant number if the isolates.


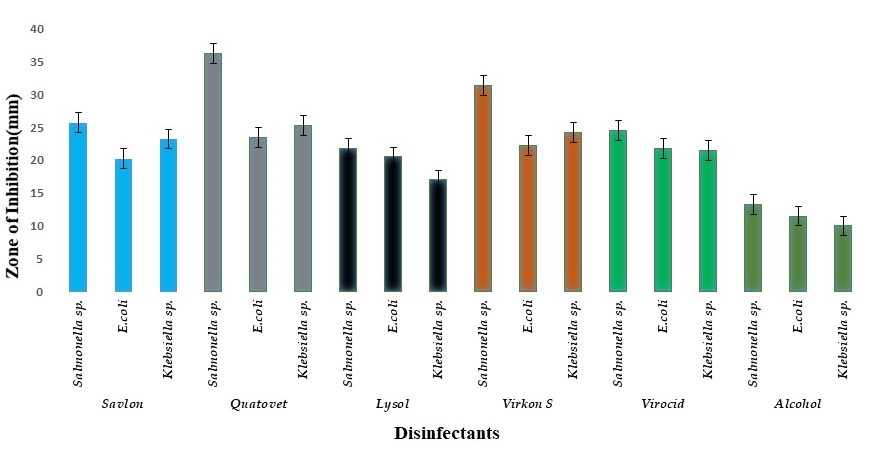


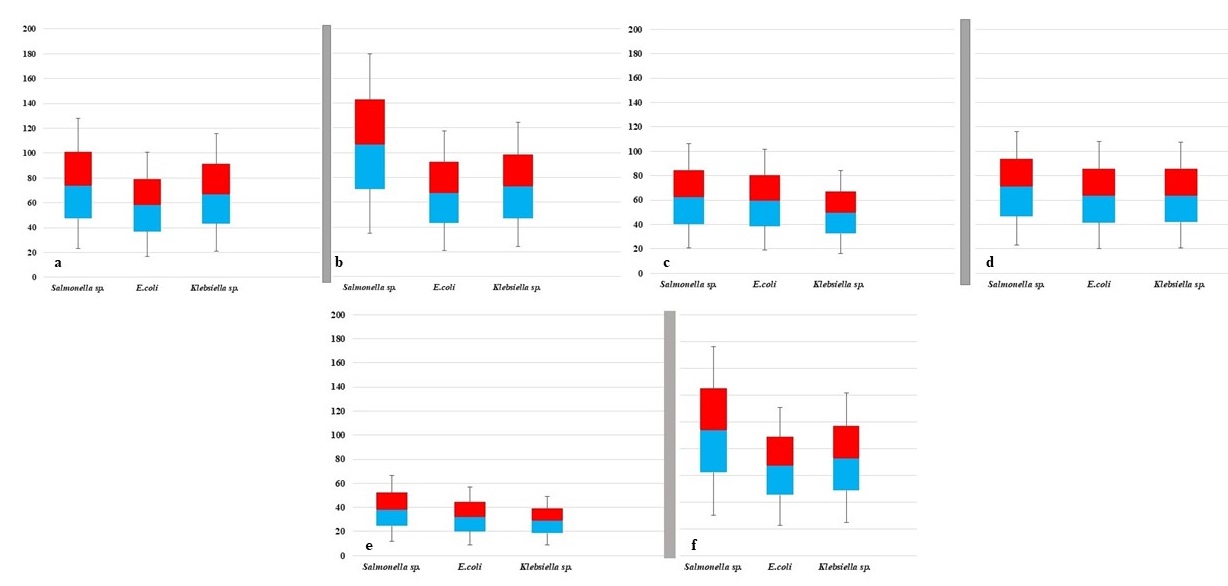
Supplementary Figure 3. Diagrammatic representation in vitro efficacy of disinfectant (100% conc.) against gram negative bacteria. Y axis represents the diameter of inhibition zone. Blue indicated Savlon, Gray Quatovet, black represented the Lysol, orange virocid and green show Alcohol.

Supplementary Figure 4. Box and Whisker plot (min/max, lower/upper quartiles and median) showing overall disinfection efficacy of various disinfectants against gram negative bacteria-axis shows most common zone of diameter area. a) Lysol; b) Quatovel; c) Lysol; d) Virocid; e)Alcohol; f) Virkon S


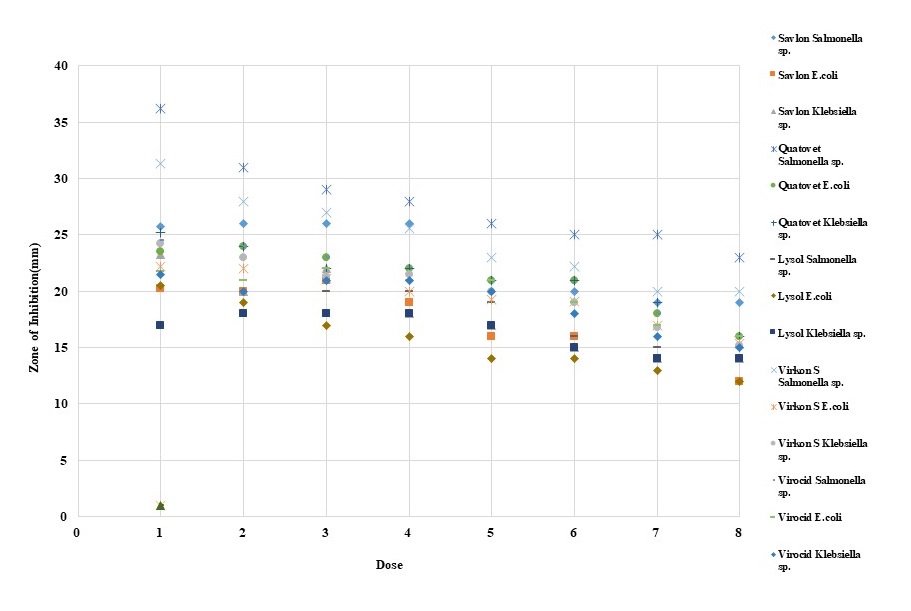


Supplementary Figure 5. Percentage of resistance of isolated gram negative microorganisms against Specific disinfectant at 100% to 30% dose Conc; X axis represent the dose percentages. Y axis represented the zone of inhibition in mm.

Supplementary Table 1. Antimicrobial susceptibility of the isolates as suggested by CLSI guideline

| **Sample ID** | **AMP** | **PG** | **TE** | **DO** | **F** | **CIP** | **NA** | **Lux** | **IMP** | **FEP** | **GN** | **S3** | **C** | **E** | **Atm** | **Tm** | **No. of R** | **Biofilm(OD)** |
| --- | --- | --- | --- | --- | --- | --- | --- | --- | --- | --- | --- | --- | --- | --- | --- | --- | --- | --- |
| SL5 | R | S | R | R | R | S | R | R | S | S | S | S | I | R | S | R | 8 | 0.28 |
| SL1 | R | S | R | R | S | S | R | R | S | S | S | S | I | R | S | S | 6 | 0.106 |
| SL10 | R | S | R | R | R | I | S | I | S | S | S | S | I | I | S | S | 4 | 0.034 |
| SL14 | R | S | S | R | R | I | R | I | S | S | S | R | I | I | I | S | 5 | 0.0273 |
| SL9 | R | R | R | R | R | I | S | R | S | S | S | R | I | I | I | R | 8 | 0.31 |
| SL29 | R | R | S | S | R | S | S | R | S | S | S | S | S | S | I | R | 5 | 0.027 |
| SL40 | S | S | R | S | R | S | S | I | S | S | S | R | S | S | S | S | 3 | 0.035 |
| SL19 | S | R | S | R | R | I | R | S | S | S | S | S | S | S | S | S | 4 | 0.033 |
| SL12 | R | R | S | S | S | S | S | S | S | S | S | S | R | R | I | R | 5 | ND |
| SL37 | S | S | R | S | R | R | R | S | S | S | S | R | R | R | I | S | 7 | 0.24 |
| SL41 | S | R | R | R | R | R | R | S | S | S | S | S | R | R | S | S | 8 | 0.21 |
| SL28 | R | S | S | R | R | S | S | S | S | S | S | R | R | R | S | S | 6 | 0.062 |
| SL30 | R | R | R | S | S | R | R | R | S | S | S | S | R | R | S | S | 8 | 0.132 |
| SL34 | S | S | R | R | S | S | S | I | S | S | S | S | R | S | I | S | 3 | 0.041 |
| SL15 | S | S | R | R | S | S | R | S | S | S | S | S | R | I | S | S | 4 | ND |
| SL17 | R | R | S | R | S | S | R | S | S | S | S | R | R | R | S | R | 8 | 0.13 |
| ML9 | S | R | S | R | R | S | S | R | S | S | S | S | R | I | S | R | 6 | 0.27 |
| ML7 | R | S | R | R | R | S | S | I | S | S | S | R | S | S | S | S | 5 | 0.104 |
| ML3 | S | S | S | R | S | I | R | I | S | S | S | S | I | S | S | S | 2 | 0.028 |
| MA1 | S | R | R | R | R | S | R | S | S | S | S | R | R | S | S | S | 7 | 0.173 |
| MA4 | R | R | S | R | R | S | S | S | S | S | S | S | R | R | S | S | 6 | 0.053 |

Here, (AMP=ampicillin; PG=Penicillin, Te=Tetracycline, Do=Doxycycline, F=Nitrofurantoin, CIP=Ciprofloxacin, Na=Nalidixic acid, Lev=Levofloxacin, IPM=Imipenem, EFP= Cefeprime, CN=Gentamycin, S3=Sulfonamide, C=Chloramphenicol, E=Erythromycin, Atm=Azithromycon, Tm=Trimethoprim. R=Resistant; I=Intermediate, S=Sensitive. OD=Optical Density of biofilm assay.

Supplemental Table 2: Zone of inhibition size (Mean±SD) Descriptive statistics for of the tested gram negative organisms by for the disinfectants comparison at 100% ConC.

| Organism | Savlon | Virocid | Lysol | Virkon S | Quatovet | Alcohol |
| --- | --- | --- | --- | --- | --- | --- |
| *Salmonella* spp*.* | 25.75±  0.96 | 24.5±  0.09 | 21.75±  0.102 | 31.25±  0.16 | 36.25±  0.0009 | 13.25±  0.03 |
| *E.coli* | 20.25±  0.02 | 21.75±  0.88 | 20.5±  0.85 | 22±  0.32 | 23.5±  0.65 | 11.5±  0.03 |
| *Klebsiella* spp. | 23.25±  0.33 | 21.5±  1.00 | 17±  0.01 | 24.25±  0.05 | 25.25±  0.09 | 10±  0.004 |
